# Synergistic Effect of Geranylgeranyltransferase Inhibitor, GGTI, and Docetaxel on the Growth of Prostate Cancer Cells

**DOI:** 10.1155/2012/989214

**Published:** 2011-06-09

**Authors:** Bin Han, Naohiro Fujimoto, Mizuki Kobayashi, Tetsuro Matsumoto

**Affiliations:** Department of Urology, University of Occupational and Environmental Health, 1-1 Iseigaoka, Yahatanishi, Kitakyushu 807-8555, Japan

## Abstract

Most advanced prostate cancers progress to castration resistant prostate cancer (CRPC) after a few years of androgen deprivation therapy and the prognosis of patients with CRPC is poor. Although docetaxel and cabazitaxel can prolong the survival of patients with CRPC, inevitable progression appears following those treatments. It is urgently required to identify better or alternative therapeutic strategies. The purpose of this study was to confirm the anti-cancer activity of zoledronic acid (Zol) and determine whether inhibition of geranylgeranylation in the mevalonate pathway could be a molecular target of prostate cancer treatment. We examined the growth inhibitory effect of Zol in prostate cancer cells (LNCaP, PC3, DU145) and investigated a role of geranylgeranylation in the anticancer activity of Zol. We, then, evaluated the growth inhibitory effect of geranylgeranyltransferase inhibitor (GGTI), and analyzed the synergy of GGTI and docetaxel by combination index and isobolographic analysis. Zol inhibited the growth of all prostate cancer cell lines tested in a dose-dependent manner through inhibition of geranylgeranylation. GGTI also inhibited the prostate cancer cell growth and the growth inhibitory effect was augmented by a combination with docetaxel. Synergism between GGTI and docetaxel was observed across a broad range of concentrations. In conclusion, our results demonstrated that GGTI can inhibit the growth of prostate cancer cells and has synergistic effect with docetaxel, suggesting its potential role in prostate cancer treatment.

## 1. Introduction

In about 80% of men with advanced metastatic prostate cancer, androgen deprivation therapy leads to improvement of symptoms and reduction of prostate specific antigen level. However, prostate cancer cells progress to castration-resistant prostate cancer (CRPC) in the vast majority of patients. Docetaxel-based chemotherapy, for the first time, demonstrated a prolongation of survival in patients with CRPC [1, 2]. Therefore, a combination of docetaxel and prednisone is current standard chemotherapy for CRPC. However, inevitable progression occurs after docetaxel treatment. TROPIC, a phase III clinical trial, demonstrated survival advantage of cabazitaxel in patients who failed prior docetaxel therapy. The median survival in cabazitaxel- treatment group, however, was 15.1 months [3], and almost all will progress. Thus, it is still urgently required to identify better or alternative therapeutic strategies for improving treatment outcome.

Bisphosphonates reduce skeletal complications in advanced malignant diseases including prostate cancer [4]. In addition, accumulated evidence has demonstrated that bisphosphonates have direct anti-cancer activities. Bisphosphonates are accumulated in the bone [5, 6] and high concentrations may be achieved in the bone. However, the concentrations of bisphosphonates in the extra-bone tissues are very low. Anti-cancer activities of bisphosphonates may be insufficient in cancers in the extra-bone tissues. In addition, Zol causes serious adverse events like jaw osteonecrosis and renal failure in some patients [7]. Bisphosphonates have those limitations despite their potential role in cancer treatment. To explore novel active agents, investigation of anti-cancer activity of bisphosphonates may be an useful strategy. Bisphosphonates exert the cellular activities by interference with the mevalonate pathway. In this pathway, small GTPases such as Ras, Rho, or Rac are modified with isoprenoid lipids, farnesyl pyrophosphate or geranylgeranyl pyrophosphate, for proper cellular localization and biological function. This post-translational lipid modification, prenylation, is performed by farnesyltaransferase or geranylgeranyltransferase (GGT). Prenylated GTPases play a pivotal 4 role in malignant transformation and contribute to the inhibition of apoptosis, and the induction of cell growth, invasion, and angiogenesis [6, 8]. Although prevention of protein prenylation may be an effective strategy for cancer treatment, its effects on prostate cancer are largely unknown.

The purpose of this study was to confirm the anti-cancer activity of zoledronic acid (Zol), one of the most potent bisphosphonates, in androgen-sensitive and -independent prostate cancer cells and evaluate the potential of geranylgeranyltransferase inhibitor (GGTI) with or without docetaxel as a treatment option for advanced prostate cancer. 

## 2. Material and Methods

### 2.1. Prostate Cancer Cells and Agents

Androgen sensitive prostate cancer cell line, LNCaP, and androgen-independent prostate cancer cell lines, PC3 and DU145, were maintained in RPMI1640, MEM, and DMEM (Sigma Chemical Co, St. Louis, MO), respectively. Those media were supplemented with 10% fetal calf serum and 1% penicillin/streptomycin (Gibco, Scotland, UK).

Zol was purchased from Novartis Pharma (Basel, Switzerland) and dissolved in sterile water containing 1% albumin. Docetaxel was purchased from Sanofi Aventis (Tokyo, Japan). Geranylgeraniol (GGOH; an analogue of geranylgeranyl pyrophosphate) was purchased from Sigma-Aldrich Japan (Tokyo, Japan) and dissolved in 100% ethanol. Geranylgeranyltransferase inhibitor (GGTI)-2147 was purchased from Calbiochem (Darmstadt, Germany) and dissolved in dimethyl sulfoxide. 

### 2.2. Cell Growth Assay

Cells (2 × 10^3^) were seeded into each well of a 96-well plate and incubated for 24 h in a humidified environment containing 5% CO_2_ at 37°C to allow the cells to attach to the plate. Following attachment, the medium was aspirated and the cells were treated with 6 × 10^−7^ M to 3 × 10^−5^ M of Zol or 1 × 10^−6^ M to 1 × 10^−5^ M of GGTI for 72 h. The control medium was exactly the same as the test medium but did not contain Zol nor GGTI. The cell number was counted before (day 0) and after drug treatment (day 3) using MTS 3-(4,5-dimethylthiazol-2-yl)-5-(3-carboxymethoxyphenyl)-2-(4-sulfophenyl)-2H-tetrazolium, CellTiter 96 Assay (Promega, Madison, WI), according to the manufacturer's instructions. Briefly, 20 *μ*l of CellTiter 96 solution were added to each well of the plate. After 60 minutes of incubation, the optical density of each sample was measured at a wavelength of 490 nm. The experiment was performed in triplicate and was replicated at least three times. 

### 2.3. Antagonism of GGOH against Zol

To examine the effect of GGOH on the growth inhibitory effect of Zol, cells were treated with 30 *μ*M of GGOH in the presence or absence of Zol (2 × 10^−5^ M). After 72 h incubation, the cell growth was determined. The experiment was performed in triplicate and was replicated at least three times. 

### 2.4. Analysis of Combined Effect of GGTI and Docetaxel

To assess the combined effect of GGTI and docetaxel, cells (3 × 10^3^) were exposed to 5 × 10^−7^ M to 1 × 10^−5^ M of GGTI and 1 × 10^−10^ M to 1 × 10^−9^ M of docetaxel. After 72 h incubation, the cell growth was determined and used to analyze the combination effect.

 Synergism of drug combination was evaluated by isobologram and combination index (CI) based on the multiple drug-effect equation of Chou-Talalay model [8] using the CalcuSyn software (Biosoft, Ferguson, MO). The isobologram method is formed by selecting a desired fractional cell kill (Fa) and plotting the individual drug doses required to generate that Fa on their respective *x*- and *y*-axes. A straight line is then drawn to connect the points. The observed dose combination of the two agents that achieved the particular Fa is then plotted on the isobologram. Combination data points that fall on the line represent an additive drug-drug interaction, whereas data points that fall below or above the line represent synergism or antagonism, respectively. The CI method is a mathematical and quantitative representation of a two-drug pharmacologic interaction. In this method, a CI less than 0.9, 0.9–1.1, and greater than 1.1 indicates that the combination effect is synergistic, additive, and antagonistic, respectively. 

### 2.5. Statistical Analysis

Groups were compared using the analysis of variance, and Tukey's test was used as the post hoc test. *P* < .05 was considered statistically significant. 

## 3. Results

### 3.1. Growth Suppression by Zol

**Figure 1:**
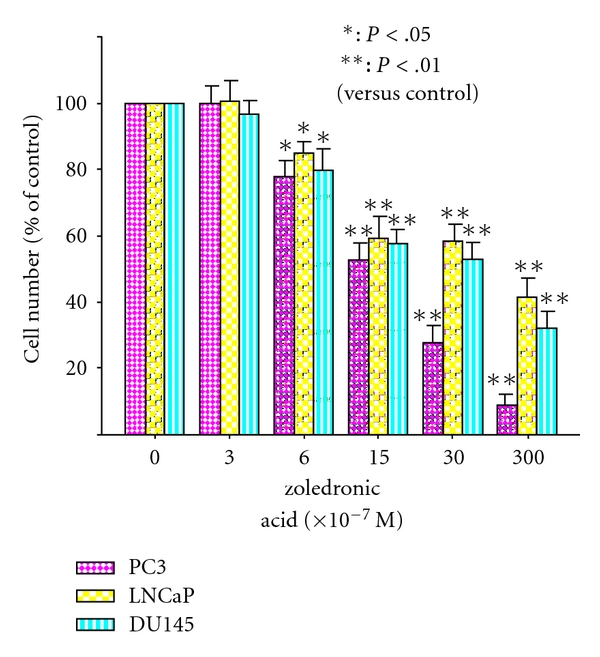
Growth inhibitory effect by Zol. Cells were incubated in the presence of Zol for 72 h, and the cell number was determined. The cell number counted in the vehicle control was defined as being equal to 100.

The cell growth assays showed that Zol inhibited the growth of prostate cancer cells in a dose-dependent manner (Figure 1). 

**Figure 2:**
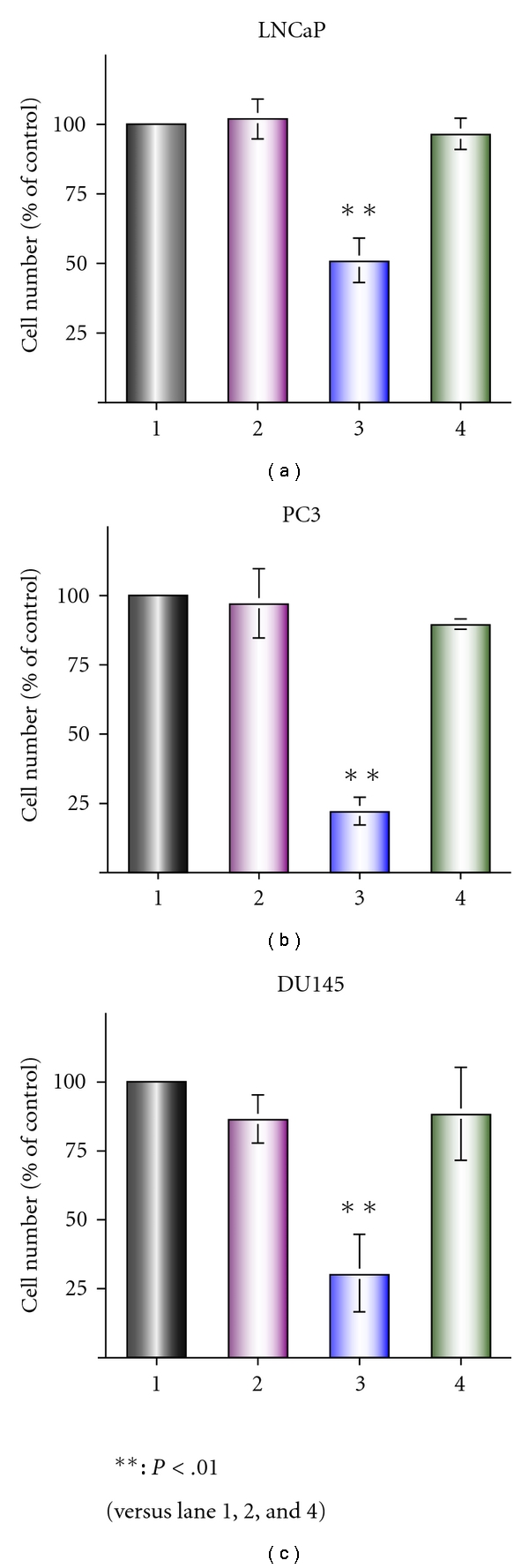
GGOH restored the growth inhibition induced by Zol. Cells were incubated in 2 × 10^−5^ M of Zol in combination with 30 *μ*M of GGOH for 72 h. The cell number counted in the vehicle control was defined as being equal to 100. Lane 1: control, lane 2: GGOH, lane 3: Zol, and lane 4: Zol + GGOH.

Bisphosphonates exert biological activities by inhibition of the synthesis of farnesyl pyrophosphate and geranylgeranyl pyrophosphate, and inhibition of geranylgeranylation seems to be important for the activity of Zol [9, 10]. To evaluate a role of inhibition of geranylgeranylation, we investigated whether replenishing cells with geranylgeranyl pyrophosphate analogue, GGOH, could reverse the growth inhibitory effect induced by Zol. Although GGOH itself did not affect the growth, it almost completely restored the growth inhibitory effect induced by Zol (Figure 2). These observations suggest that geranylgeranylation seems to be the main target of Zol in prostate cancer cells. 

### 3.2. Growth Suppression by GGTI and Synergism with Docetaxel

**Figure 3:**
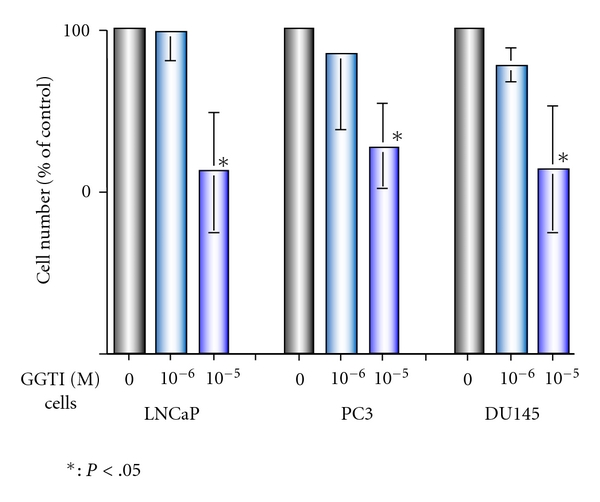
Growth inhibitory effect of GGTI. Cells were incubated in 1 *μ*M–10 *μ*M of GGTI for 72 h, and the subsequent cell growth was determined. The cell number counted in the vehicle control was defined as being equal to 100.

We investigated the effect of GGTI, a specific inhibitor of geranylgeranylation, on the growth of prostate cancer cells. As shown in Figure 3, 10 *μ*M of GGTI inhibited the growth of LNCaP, PC3, and DU145 cells by 45%, 37%, and 44%, respectively (*P* < .05 versus control). These results suggest that geranylgeranylation could be a molecular target and that GGTI appears to be a potential agent for the treatment of prostate cancer. 

**Figure 4:**
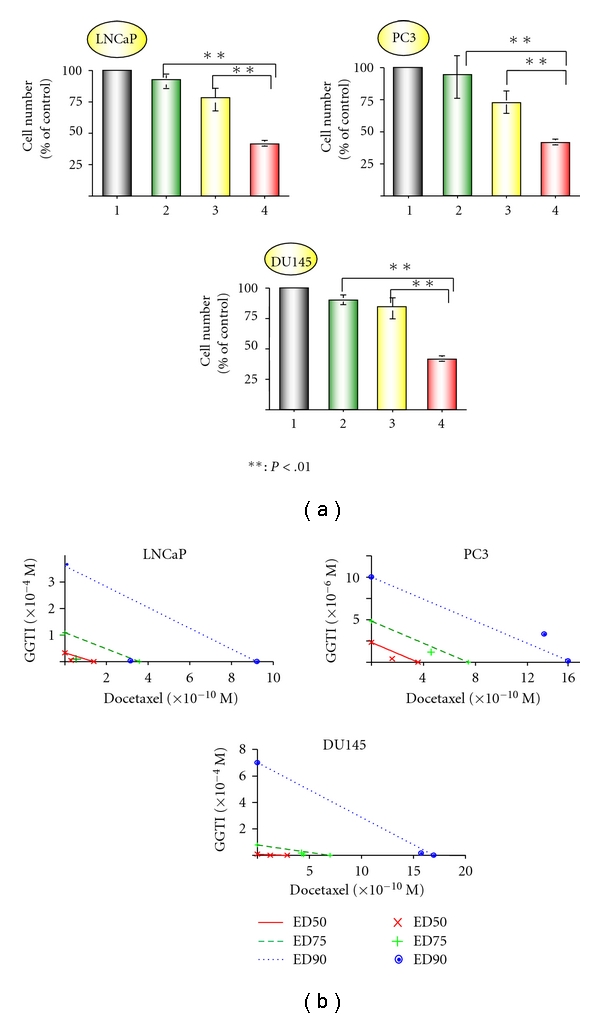
Combined effect of GGT and docetaxel. (a) Cells were exposed to GGTI (1 *μ*M) in combination with docetaxel (10^−10^ M). Following 72 h incubation, the cell growth was determined. Lane 1: control, lane 2: GGTI, lane 3: docetaxel, and lane 4: GGTI and docetaxel. (b) Cells were incubated in the serial concentrations of GGTI, docetaxel, or combination of those agents for 72 h. Cell growth was determined, and combination effect was analyzed by isobologram. The individual doses of GGTI and docetaxel to achieve 90% (dotted line) growth inhibition (Fa = 0.90), 75% (hyphenated line) growth inhibition (Fa = 0.75), and 50% (straight line) growth inhibition (Fa = 0.50) were plotted on the *x*- and *y*-axes. Combination index values are represented by points above (indicate antagonism between drugs) or below the lines (indicate synergy). (X symbol) ED_50_, (plus sign) ED_75_, and (closed circle) ED_90_.

Since anti-cancer activities of bisphosphonates can be augmented by cytotoxic agents [9–12] and we have observed the combination effect of Zol and docetaxel (data not shown), we examined whether docetaxel can also enhance the effect of GGTI. As shown in Figure 4(a), the combination of GGTI and docetaxel showed significantly stronger effect than each drug alone. Regarding combination effect, isobolograms were constructed for Fa values of 0.50, 0.75, and 0.90, representing 50%, 75%, and 90% growth inhibition, respectively. All combination data points were below the line except Fa 0.9 in PC3 cells (Figure 4(b)). 

CIs of GGTI and docetaxel were less than 0.9 at concentrations of Fa 0.02 to 0.95, 0.02 to 0.75, and 0.15 to 0.85 in LNCaP, PC3, and DU145 cells, respectively. Both isobologram and CI methods indicated synergism of GGTI and docetaxel across a broad range of concentrations in all three prostate cancer cell lines. 

## 4. Discussion

In addition to their effects on the bone, accumulating evidence has suggested that nitrogen-containing bisphosphonates, including Zol, have anti-cancer activities in a variety of cancer cells [6, 7]. In the present study, we confirmed the growth suppression induced by Zol in androgen-sensitive and -independent prostate cancer cells. Bisphosphonates are accumulated and retained in the bone for a long time with a half-life of about 200 days [13]. Thus, a high tumoricidal concentration and the long-lasting accumulation of bisphosphonates in the bone may contribute to their efficacy against tumors located in bone tissues [6]. However, the concentrations of bisphosphonates in the extraosseous tissues are much lower than the effective concentrations *in vitro.* For example, following the intravenous administration of a conventional dose (4 mg) of Zol, concentrations of higher than 10^−6^ M (required to exert a direct anti-cancer activity) is hardly achievable in the plasma. Zol of higher concentrations than 10^−6^ M remains in the plasma only shorter than 1 h after injection [5]. Therefore, sufficient concentrations for anti-cancer activity are hardly achievable in cancers in the extra-osseous tissues. Thus, we investigated the mechanisms of the anti-cancer activities induced by Zol to explore the other active agents. In the present study, we demonstrated that replenishing the cells with GGOH, which restores geranylgeranylation, can overcome the effects of Zol and that GGTI can inhibit the growth of prostate cancer cells. Goffinet et al. [9] and Coxon et al. [10] showed that Zol inhibited the growth and induced apoptosis in prostate cancer cells through the inhibition of geranylgeranylation. Those observations indicate that the inhibition of geranylgeranylation plays a pivotal role in the growth inhibition and the induction of apoptosis and that geranylgeranylation appears to be a molecular target of prostate cancer treatment. GGT catalyzes protein geranylgeranylation, which is critical for function of proteins. GGT substrates include Ras, Rac, and Rho GTPases and the *γ*-subunits of most heterotrimeric G-proteins [11]. Inhibition of GGT by GGTI can inactivate CDK2/4 through the p21/p15 kinase inhibitors downstream of Rho, resulting in cycle arrest at G_0_/G_1_ [12, 14]. GGTI can also stimulate induction of apoptosis in both normal [15, 16] and transformed cell lines including prostate cancer cells [10, 17, 18]. GGTI also regulates cytoskeletal integrity and motility of prostate cancer cells [19]. Those observations raise the possibility of GGTI as a useful agent for the management of prostate cancer. 

The antiproliferative activity of bisphosphonates, including Zol, is augmented by anticancer agents like doxorubicin, taxanes, etoposide, cisplatin, irinotecan, or imatinib in prostate, breast [20, 21], lung [22], or bladder cancer cells [23]. Thus, we investigated whether the growth inhibitory effect of GGTI was also augmented by docetaxel, a standard cytotoxic agent for prostate cancer. As a result, the growth inhibitory effect of docetaxel was augmented by docetaxel, and we demonstrated for the first time that a combination effect of GGTI and docetaxel is synergistic. Inhibition of geranylgeranylation prevents the prenylation of Rac1, resulting in decreased Rac1 activity. Docetaxel also prevent Rac1 activation [24]. Therefore, GGTI and docetaxel may work synergistically. However, the mechanisms by which GGTI enhances the effect of docetaxel are largely unknown and remain to be elucidated.

Taken together, GGTI with or without docetaxel may be an useful treatment strategy for patients with CRPC, even after progression on docetaxel-regimens. Recently, the Phase 1 clinical trial of GGTI (GGTI 2418) was initiated in patients with metastatic solid tumors for which standard treatments have failed, or for whom standard therapies are not available, and its safety and tolerability will be evaluated.
